# Role of Flooding Patterns in the Biomass Production of Vegetation in a Typical Herbaceous Wetland, Poyang Lake Wetland, China

**DOI:** 10.3389/fpls.2020.521358

**Published:** 2020-10-16

**Authors:** Xue Dai, Zhongbo Yu, Guishan Yang, Rongrong Wan

**Affiliations:** ^1^State Key Laboratory of Hydrology–Water Resources and Hydraulic Engineering, Hohai University, Nanjing, China; ^2^College of Hydrology and Water Resources, Hohai University, Nanjing, China; ^3^Key Laboratory of Watershed Geographic Sciences, Nanjing Institute of Geography and Limnology, CAS, Nanjing, China; ^4^University of Chinese Academy of Sciences, Beijing, China

**Keywords:** end-of-season biomass, inundation depth, inundation duration, flooding pattern, wetland, Three Gorges Dam

## Abstract

Flooding is an important factor influencing the biomass production of vegetation in natural wetland ecosystems. However, how biomass production is linked to flooding patterns in wetland areas remains unclear. We utilized gauging station data, a digital elevation model, vegetation survey data, and a Landsat 8 image to study the effects of average inundation depth (AID) and inundation duration (IDU) of flooding on end-of-season biomass of vegetation in Poyang Lake wetland, in particular, after operation of Three Gorges Dam. The end-of-season biomass of wetland vegetation showed Gaussian distributions along both the AID and IDU gradients. The most favorable flooding conditions for biomass production of vegetation in the wetland had an AID ranging from 3.9 to 4.0 m and an IDU ranging from 39% to 41%. For sites with a lower AID (<3.9 m; IDU < 39%), the end-of-season biomass values were positively related, whereas for sites with a higher AID (4.0 m; IDU > 41%), the end-of-season biomass values were negatively related. After the operation of the Three Gorges Dam, flooding patterns characterized by AID and IDU of the Poyang Lake wetland were significantly alleviated, resulting in a mixed changing trend of vegetation biomass across the wetland. Compared with 1980–2002, the increase of end-of-season biomass in lower surfaces caused by the alleviated flooding pattern far exceeded the decrease of end-of-season biomass in higher surfaces, resulting in an end-of-season biomass increase of 1.0%–6.7% since 2003. These results improved our understanding of the production trends of vegetation in the wetland and provided additional scientific guidance for vegetation restoration and wetland management in similar wetlands.

## Introduction

Plants are the primary producers in wetland ecosystems, providing food and habitat for many species of fish, amphibians, water birds, and other forms of life (Fisher and Willis, 2000; Kingsford and Thomas, 2004; Xie et al., 2015). Therefore, changes in the biomass production of wetland vegetation often affect the dynamics of the entire wetland (Tockner and Stanford, 2002; Paillisson et al., 2006; Gathman and Burton, 2011; Jia et al., 2017). In addition, biomass production is the main carbon input in wetland ecosystems, thus serving as a crucial component of carbon sequestration in the earth’s carbon cycle (Kayranli et al., 2010; Erickson et al., 2013). In seasonally inundated herbaceous wetlands, however, which are affected by non-stationary flooding patterns, biomass production of vegetation usually changes rapidly and shows strong spatial heterogeneity (Poff et al., 1997; Dornova, 2012; Byrd et al., 2014; Watson and Corona, 2018).

Flooding patterns affect vegetation production of wetlands through multiple aspects, including the timing (e.g., inundation duration) and magnitude (e.g., inundation depth) of flood events (Visser et al., 2006; Wantzen et al., 2008; Li et al., 2012). Moreover, the impacts of flooding patterns on vegetation productivity are complex. They may stimulate or inhibit vegetation production depending on such factors as the adaptability of plants to flood disturbances and other abiotic factors like elevation, slope, and reallocation of sediments (Casanova and Brock, 2000; Mokrech et al., 2017). For example, in wetlands experiencing extreme floods, mechanical damages caused by flooding can directly reduce plant biomass and can even lead to the complete disappearance of aboveground parts of plants (Dai et al., 2016; Wan et al., 2018). Vegetation restoration after extreme flood disturbances often takes several years (Kingsford, 2000; Nechwatal et al., 2008). In contrast, the abundant water resources and soil nutrients introduced by floods can optimize the growth conditions of vegetation, thus promoting increased productivity for some sites (Burke et al., 1999, 2003; Clawson et al., 2001). Trade-offs in site specific impacts from flooding are difficult to quantify at the landscape scale, yet the net balance of consequences is important for continued management of these diverse ecosystems. This is particularly true in large, heterogeneous ecosystems, such as the Poyang Lake wetland, China (Sang et al., 2014).

Poyang Lake is the largest freshwater lake and area of alluvial wetlands in China (You et al., 2017; Wan et al., 2018). The extensive and prolonged summer flooding has characterized the water regime of the wetland (Liu Y. B. et al., 2013; Zhang et al., 2014). The average water levels of the summer flood season and the drier part of the year differ by about 11 m, and the duration of these two periods in a given year is about 33% and 66%, respectively (Dai et al., 2015). The vast, seasonally inundated alluvial plain has developed to be the largest freshwater wetland in China, of which the average area is about 3000 km^2^ (Liu C. L. et al., 2013; Dai et al., 2016). In recent decades, the operation of the Three Gorges Dam upstream of the lake’s mouth has significantly altered the water regimes of the Poyang Lake, in particular, in regard to its flooding patterns (Hu et al., 2007; Mei et al., 2016). Consequently, many studies have revealed the significant shift in the expansion and contraction of flooding events and their influence on vegetation distribution and the habitat of migratory birds (Barzen et al., 2009; Tan et al., 2015; Xu et al., 2015). For example, our previous studies found that the premature surface exposure time in the Poyang Lake wetland after the Three Gorges Dam dramatically stimulated the expansion of vegetation (Dai, 2018; Wan et al., 2018). It is mainly because that the earlier recession of summer floods prolonged the vegetation growth period in autumn to a certain extent (Dai et al., 2016, 2019). Even though these studies have elucidated the changes of the ecohydrological environment in the Poyang Lake wetland after operation of the Three Gorges Dam, the relationship between biomass production of vegetation and flooding patterns in this wetland area remains largely unclear.

This study examined the impacts of changing flooding patterns in Poyang Lake on its biomass production of vegetation. Specifically, we used two variables of flooding—that is, inundation duration (IDU) and average inundation depth (IDU)—to describe the flooding pattern of the wetland and used the end-of-season biomass quantified using NVDI^∗^biomass calibrated relationships to describe the vegetation biomass production of the wetland. Our specific objectives were as follows: (i) describe and quantify the patterns of vegetation biomass production across major flooding gradients in the extensive wetland, and (ii) reveal the effects of changing flooding conditions on the vegetation biomass production of the wetland since the operation of the Three Gorges Dam.

## Materials and Methods

### Study Area

**FIGURE 1:**
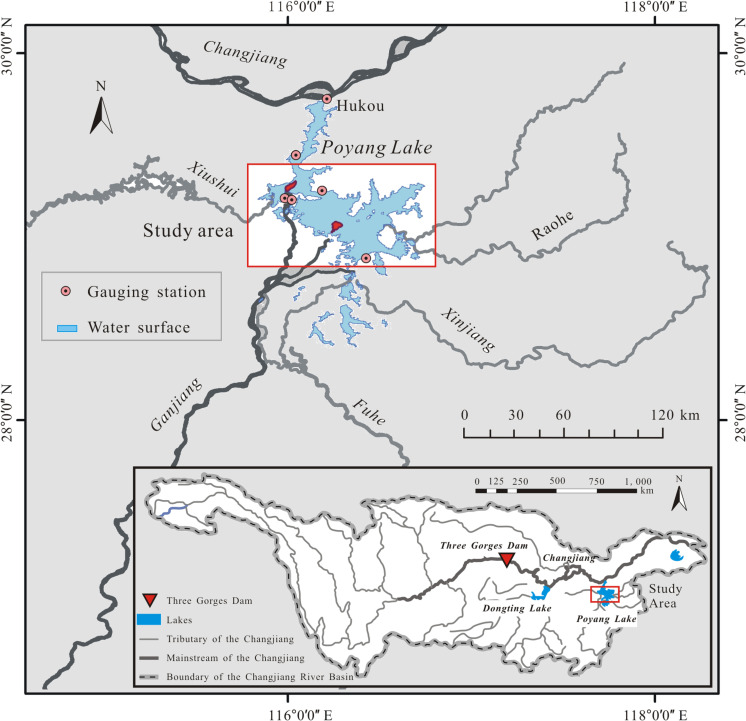
Map of the Poyang Lake wetland with insert showing its location south of the Changjiang River.

Poyang Lake (115°49′E–116°46′E, 28°24′N–29°46′N) (Figure 1) receives discharge from the give upstream rivers (Ganjiang, Fuhe, Xinjiang, Raohe, and Xiushui) and flows into the Changjiang River from the lake mouth (Hukou). Influenced by the inflow of the five upstream rivers and the block effect of the Changjiang River on its outflow, the summer flood generally lasts a prolonged period from June to September (Hu et al., 2007; Zhang et al., 2014; Dai et al., 2015). The prolonged summer flood provides an inability for the trees to survive in this wetland. Thus, Poyang Lake wetland is a typical herbaceous wetland with a relatively high vegetation homogeneity (Wu et al., 2012; Sang et al., 2014). Specifically, over 80% of its vegetated area is covered by *Carex* spp., and the left 20% is mainly covered by *Phragmites* spp. (You et al., 2017; Dai et al., 2019). In the flood seasons, almost all floodplains are inundated, and the coverage area of the water surface reaches more than 3,000 km^2^. In the drier part of a given year (October to April of the following year), the lake stage decreases and eventually recedes largely into channels created by its tributaries and shallow depressions. At that point, the water surface covers less than 1,000 km^2^ (Liu Y. B. et al., 2013; Wan et al., 2018). The extensive exposed lakebed turns into vegetated habitats, which cover more than two-thirds of the lakebed. Because of the flat terrain and fertile sediment, the alluvial plains are highly productive habitats and provide many ecosystem services, including sediment stabilization, habitat and biodiversity, water quality purification, and carbon and nutrient sequestration (Liu and Nian, 2012).

### Data and Processing

#### End-of-Season Biomass Mapping in the Poyang Lake Wetland in 2016

**FIGURE 2:**
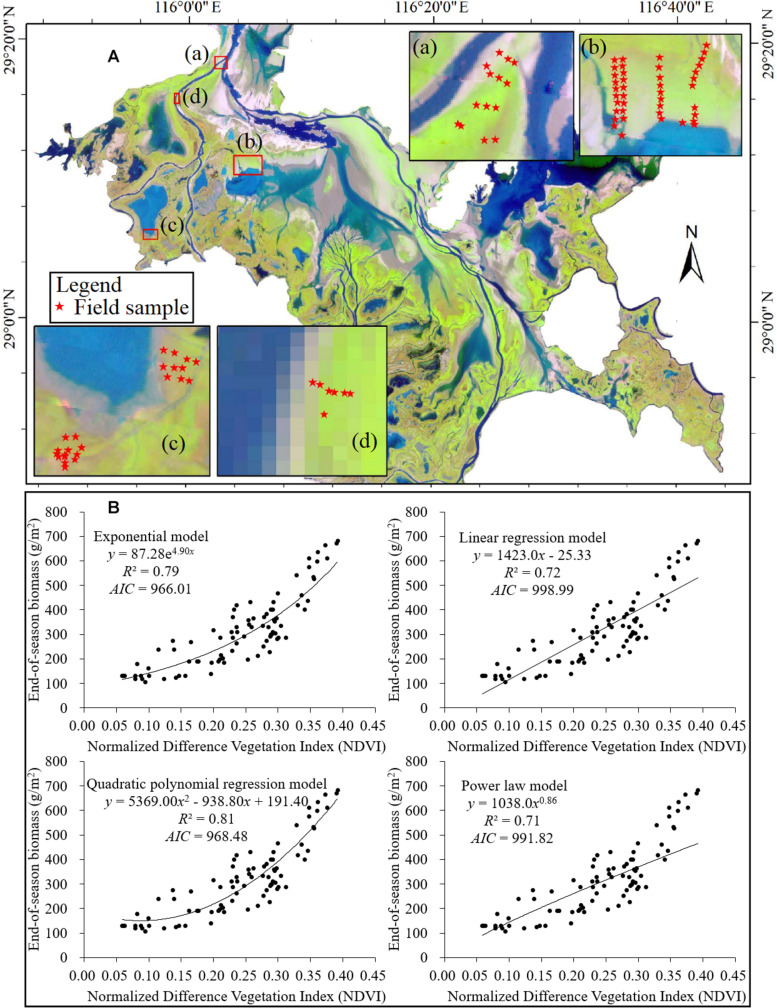
**(A)** Locations of field sampling sites (the background image is a false color composition of the Landsat OLI image with the band 5, band 4, and band 3 to red, green, and blue, respectively) and **(B)** models established for the relationship of NDVI (*x*) and end-of-season biomass (*y*) in the Poyang Lake wetland in 2016.

In this study, we mapped the end-of-season biomass of the Poyang Lake wetland in 2016 using a model of NDVI and end-of-season biomass developed based on the regression analysis between the field-observed biomass records and the remotely sensed Normalized Difference Vegetation Index (NDVI). The spatial resolution of the NDVI data is 30 m × 30 m, which was calculated from the Landsat OLI image acquired on December 16, 2016 (ID: LC08L1TP12104020161216) by the NDVI tool in ENVI 5.1. The field data was gathered during November 24th to December 3rd 2016 at 86 sites from four sub-regions of the Poyang Lake wetland (Figure 2A). At each sub-region, field samplings were conducted perpendicular to the shoreline to cover different levels of the flooding pattern gradient, and were conducted parallel to the shoreline to collect more samples. At each site, aboveground biomass in a 1 × 1 m plot was harvested by clipping the plants at soil level. Wet biomass was weighed and then converted to dry biomass through a proportionality constant, i.e., the water content factor (taken as 0.3 via sample analysis) (Dai, 2018). The geographic coordinates of those sites were recorded by a GPS (GPS 60, GARMIN), which were then used to extract the corresponding NDVI from the coincident Landsat NDVI image.

We tested four regression models, including a power law model, an exponential model, a linear regression model, and a polynomial model to fit the relationship between the field measured end-of-season biomass and its corresponding NDVI values. The ordinary least squares method estimated the model parameters and the Akaike Information Criterion (AIC) measure the relative quality of those models. After comparison, we utilized the quadratic polynomial regression model *y* = 5369.00*x*^2^ – 938.80*x* + 191.40 (*n* = 85, *R*^2^ = 0.81, *AIC* = 968.48) to estimate the end-of-season biomass of the wetland. Because the quadratic polynomial regression model has a superior performance to the other three regression models (Figure 2B).

#### Flooding Pattern Variables Mapping

We used two variables of flooding—annual average inundation depth (AID) and inundation duration (IDU)—to characterize the energy and timing of floods in this wetland. We mapped two flooding pattern variables of each year during the time period from 1980 to 2016 using the long-term daily water levels of six dispersed gauging stations (Figure 1) and a digital elevation model (DEM, with a 5 m × 5 m spatial resolution) of the Poyang Lake wetland. Both the hydrologic data and the DEM were provided by the Jiangxi Provincial Hydrological Bureau, China.

For the flooding pattern variable mapping, we first generated the daily water surface elevation series of the wetland in each year by averaging the daily water levels of the six gauging stations and rasterizing them to the whole wetland in the same resolution with the end-of-season biomass map (30 m × 30 m). Then, we resampled the DEM of the wetland to the same resolution with the water-surface elevation maps. For each pixel, by comparing the elevation of water surface and land surface, we identified whether it was inundated and how deeply it was inundated on each day. Finally, we calculated the AID as the average inundation depth in a year with a unit of meter (m); we counted IDU as the total number of submerged days in a year and then standardized the results by dividing the total length of each year. Thus, the IDU ranged from 0 to 1, with a unit of percentage of the year (%). High IDU values indicated long inundation duration, and vice visa.

#### Model the Relationships of End-of-Season Biomass and Flooding Pattern Variables

In this study, we modeled the relationship between the end-of-season biomass and each flooding pattern variable according to the Gaussian mixture model (GMM). Specifically, by matching the maps of end-of-season biomass and the two variables of the flooding patterns pixel by pixel, we obtained the averaged end-of-season biomass within each gradient of the flooding pattern variable. We set the gradient intervals for AID and IDU to 0.1 m and 0.10 (10%), respectively. Then we used GMM to fit the distribution pattern of the end-of-season biomass along the flooding pattern gradient. We applied the GMM model in this study because it is flexible enough to fit both the typical hump-shaped curves between variables and the monotonic or sigmoidal curves between variables within restricted ranges of either axis (Gomez-Aparicio et al., 2011; Ruiz-Benito et al., 2014). The general function of GMM can be expressed as follows:

y=∑i=1nci⁢e*⁢x⁢p⁢(-12⁢((x-μi)2ti2))

where *y* is the end-of-season biomass; *x* is the specific flooding pattern variable (i.e., AID and IDU); and *n* is the number of peaks of GMM model to fit, where 1 < *n* < 2 in this study. For each peak, *c*_*i*_ is the maximum of end-of-season biomass, μ_i_ is the optimum AID or IDU condition for end-of-season biomass, which appears when *y* equals *c*_*i*_; and *t*_*i*_ is related to the peak width, which reveals the most conducive range of each flooding variable for vegetation production.

#### Extract the Impacts of Changing Flooding Patterns on End-of-Season Biomass After the Three Gorges Dam

To achieve this objective, we first mapped the multi-year average AID and IDU of the wetland in two time periods: 1980–2002 (pre-Three Gorges Dam) and 2003–2016 (post-Three Gorges Dam). We characterized the variation of each variable in these two periods according to its differences in gradient interval average values. Then, we input these values into the obtained GMM models of end-of-season biomass to predict the corresponding vegetation biomass changes triggered by the variations in the flooding pattern variable.

## Results

### End-of-Season Biomass of Poyang Lake Wetland in 2016

**FIGURE 3:**
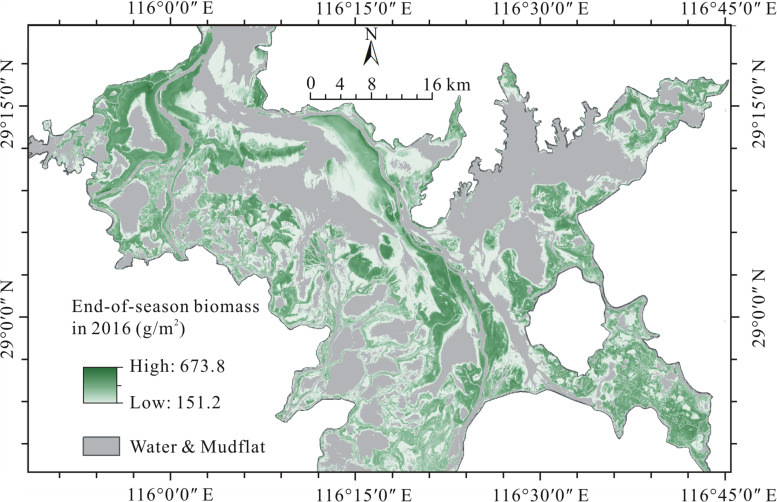
End-of-season biomass of Poyang Lake wetland in 2016.

The end-of-season biomass of Poyang Lake wetland in 2016 is shown in Figure 3. The mean end-of-season biomass of the wetland was 234.0 g/m^2^ and the standard deviation was 99.9 g/m^2^. The end-of-season biomass varied in a wide range (151.2–673.8 g/m^2^) and showed a clear spatial pattern. Specifically, low end-of-season biomass values occurred in the depressions and regions near to the main lake water surfaces; high end-of-season biomass values occurred in the moderate elevation part of the wetland; and moderate end-of-season biomass values occurred in the ridges of the wetland.

### Flooding Pattern of the Poyang Lake Wetland in 2016

**FIGURE 4:**
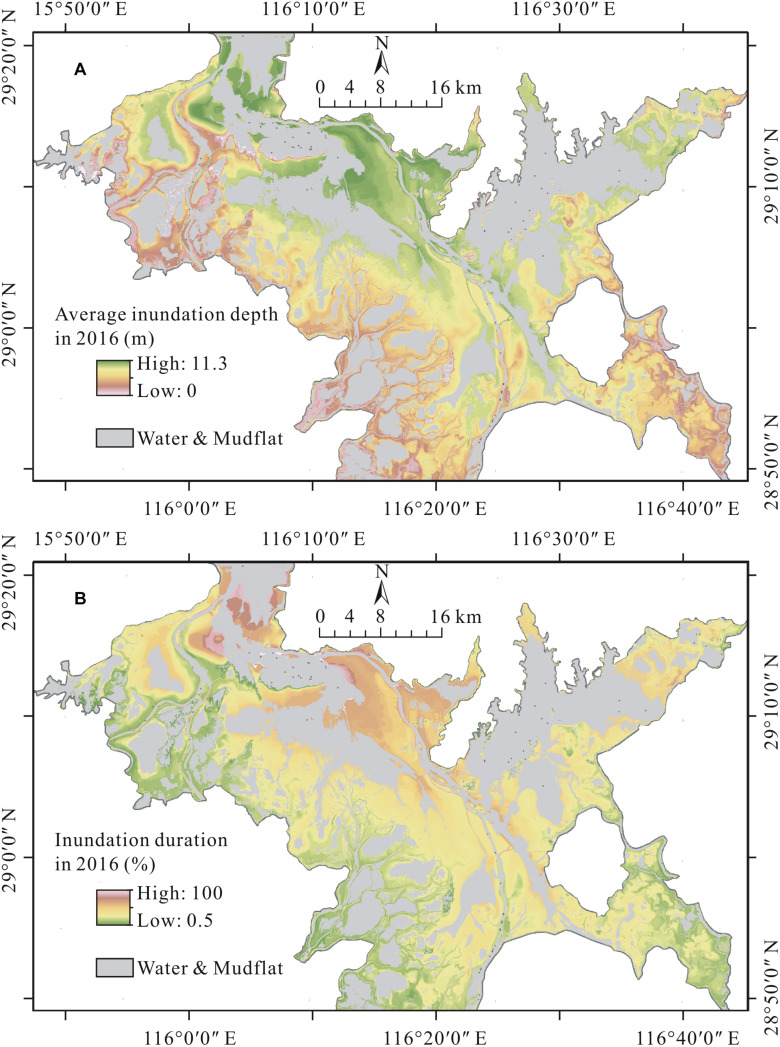
**(A)** Average inundation depth and **(B)** inundation duration of the Poyang Lake wetland in 2016.

The AID and IDU maps of the Poyang Lake wetland in 2016 are shown in Figures 4A,B, respectively. As shown in Figure 4A, the AID of the wetland ranged from 0.1 to 11.3 m in 2016, with a mean of 4.2 m and a standard deviation of 1.7 m. Most AIDs of the vegetated area in the wetland were less than 6.0 m. Moreover, the AID values varied along the distance to the water surface: AIDs exhibited larger values surrounding the deep flow channels and permanently flooded sublakes, whereas they exhibited lower values in regions near the wetland margins, such as the lake levees and river banks. Figure 4B shows that the vegetated area of the wetland was inundated, on average, for 46% of the days in 2016. The specific IDUs of different sites ranged from 1% to 80%, with a standard deviation of 16%. Similar to AID, IDU of the wetland also showed a clear spatial pattern across the wetland. Specifically, the near-shore area experienced a longer inundation, whereas the offshore area experienced a shorter inundation.

### Distribution of End-of-Season Biomass Along the Gradients of Flooding Pattern Variables

**FIGURE 5:**
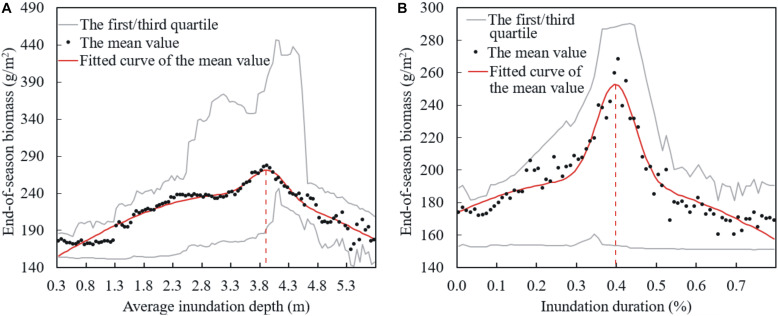
Distributions of end-of-season biomass along the gradients of **(A)** average inundation depth and **(B)** inundation duration in Poyang Lake wetland.

The values of end-of-season biomass in different AID and IDU intervals are averaged and plotted as black dots in Figure 5, along with their GMM fitting curves (the red lines). Moreover, we also plotted the first and third quartiles of the end-of-season biomass values within the same inundation variable intervals in Figure 5 (the gray lines), which show the overall distribution of the data. Table 1 summarizes the parameters of the end-of-season biomass models fitted by the GMM function via the variables of AID and IDU. It is evident that the distributions of end-of-season biomass along both the IDU and AID gradients were hump-shaped. Specifically, for AID, the most productive sites in the wetland were in the condition of an AID equal to 4.0 m (3.9 m, 4.0 m), of which the averaged end-of-season biomass peaked at about 271.0 g/m^2^ (271.2 g/m^2^, 269.4 g/m^2^). For IDU, the most productive sites would have 40% (39%, 41%) of the days inundated during the whole year. Plants experiencing the most optimal IDU condition weighed about 252.8 g/m^2^ (251.2 g/m^2^, 251.8 g/m^2^) at the end of season on average. The values of end-of-season biomass in sites with lower AID values (<4.0 m; IDU < 40%), were positively correlated with the variations of the AID (IDU), whereas the values of end-of-season biomass in sites with larger AID values (>4.0 m; IDU > 40%) were negatively correlated with the AID (IDU).

**TABLE 1:** Parameters of end-of-season biomass models fitted by GMM function via the flooding pattern variable AID and IDU.

	**AID and end-of-season biomass model**	**IDU and end-of-season biomass model**
*c*_*1*_	238.10	193.70
μ_*1*_	3.31	0.34
*t*_*1*_	4.58	1.02
*c*_*2*_	37.55	59.87
μ_*2*_	3.95	0.40
*t*_*2*_	0.43	0.07
*R*^2^	0.92	0.91

### Changes in Flooding Patterns and Their Additional Impacts on Vegetation Production

**FIGURE 6:**
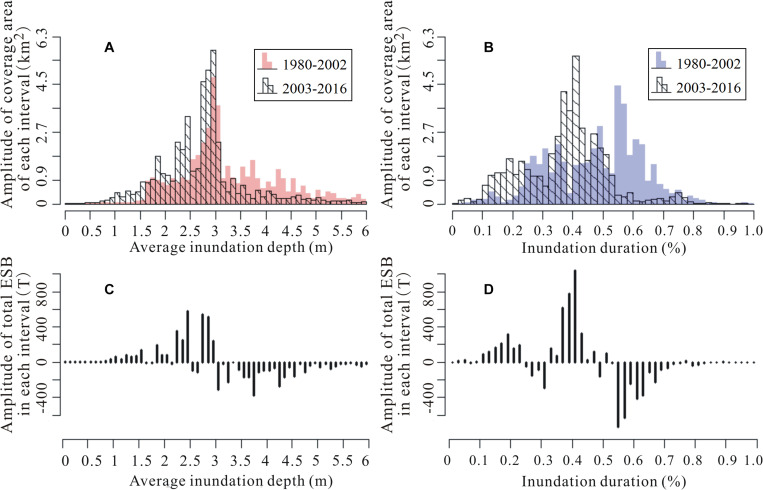
Changes in flooding patterns of Poyang Lake wetland between 1980–2002 and 2003–2016 (**A** for AID and **B** for IDU) and their additional impacts on end-of-season biomass of the wetland (**C** for AID and **D** for IDU).

The multi-year average AIDs for 1980–2002 and 2003–2016 were 3.2 m and 2.7 m, respectively (Figure 6A). The multi-year average IDUs for 1980–2002 and 2003–2016 were 70.5% and 60.0%, respectively (Figure 6B). Apparently, compared with 1980–2002, the distributions of both AID and IDU dramatically shifted to skew toward the right during 2003–2016. That is, for most sites in the wetland, the AID and IDU values tended to become lower. Given the Gaussian form response of end-of-season biomass to flooding pattern variables, the influence of alleviated AID (IDU) on end-of-season biomass varied across the extensive wetland (Figures 6C,D). Specifically, for sites in which the values of end-of-season biomass were negatively correlated with the AID (IDU)—that is, sites with higher AIDs (IDUs)—shallower inundation depth (shorter inundation duration) in 2003–2016 contributed to higher end-of-season biomass. On the contrary, for sites in which the values of end-of-season biomass were positively correlated with the AID (IDU)—that is, sites with lower AIDs (IDUs)—shallower inundation depth (shorter inundation duration) during 2003–2016 contributed to lower end-of-season biomass. Moreover, because the coverage area of sites converting from deep and long inundation (AID > 3.5 m; IDU > 54%) to be more productive far exceeded the coverage area of sites converting from shallow and short inundation (AID < 1.6 m; IDU < 24%) to be less productive, the increase of end-of-season biomass in the wetland far exceeded the decrease of that since 2003 (Figures 6A,B). Hence, although a mixed changing pattern existed for the end-of-season biomass variations across the wetland (Figure 6B), on average, the end-of-season biomass for the whole wetland increased by 2.4 g/m^2^ stimulated by the shift of AID since 2003, with an increased rate of about 1.0%. According to the prediction of the end-of-season biomass model predicted by IDU, the average end-of-season biomass of the wetland also showed an increasing trend since 2003 but with a much larger magnitude. That is, the end-of-season biomass increased by 15.7 g/m^2^ on average stimulated by the shift of IDU since 2003, with an increased rate of about 6.7%.

## Discussion

In our previous research, we discussed the response of vegetation biomass to land surface exposure time after flooding in the Poyang Lake wetland to reveal the influence of Three Gorges Dam on biomass production (Dai, 2018). After the operation of the Three Gorges Dam, the land surface of the wetland has been exposed prematurely. The land surface premature triggered by the Three Gorges Dam has been considered to have the most dramatic influence on vegetation because the main wetland vegetation sedges begin to re-grow exactly as the summer flooding in this wetland ends (Mei et al., 2016; Wan et al., 2018).

In this study, however, we found that the summer flood patterns also played an important role in vegetation production in the wetland, even though sedges generally were dormant during the summer floods. Specifically, our results revealed that with shifts in flooding duration and inundation depth after the operation of the Three Gorges Dam that biomass has on average for the full area increased. Moreover, similar Gaussian distribution patterns of the end-of-season biomass have been found for both AID and IDU gradients in this wetland, as well as the hydrologic variable-stimulated biomass production since the operation of the Three Gorges Dam. In previous studies, the intermediate disturbance hypothesis has been applied and tested in larger systems for the diversity pattern of vegetation, which believes that the diversity will likely be higher at intermediate sites because of the unidirectional variations in the disturbance (Osman, 2015). In this study, we found a biomass pattern consistent with the intermediate disturbance hypothesis but not resulting from a gradient in species disturbance. Actually, we found that gradients in flooding pattern variables such as inundation depth and inundation duration lead to the different biomass production at the extremes based on species tolerances. Our findings suggest that gradients of biomass or diversity can result not only from unidirectional variations in disturbance but from variations in one or more environmental variables.

Flooding patterns in the vegetation production of the wetland mainly affect the flooding-oriented life cycle of vegetation in the wetland (Lei et al., 2011; Wu et al., 2012; Zhang et al., 2012). Therefore, before analyzing the effects of flooding pattern variables on vegetation production, we first clarified the life cycle of plants in the wetland. In the spring, plants in both lower and higher surfaces of the wetland begin to grow promoted by an increase in soil moisture and absent submersion with an increase in the lake-water level (Sang et al., 2014; Dai et al., 2016). During the summer flood season, the rising water level spreads over the lower surfaces and eventually completely covers the plants; thus, aboveground shoots wither and the plants go dormant. Plants at higher surfaces still stand above the water surface and continue to grow (Zhang et al., 2013; Tan et al., 2015). During the autumn, the water level recedes and plants at the lower surfaces initiate growth as the sites are eventually exposed. The plants in the higher surfaces turn to a senesce period in autumn (Wan et al., 2018; Dai et al., 2019). Note that the main water stresses for plants in higher and lower surfaces are different. Plants in the higher surfaces usually have to withstand water stresses caused by extended dry phases, whereas plants in the lower surfaces usually have to face water stresses caused by extended floods. Thus, the Gaussian form effects of both AID and IDU to end-of-season biomass across the wetland can be explained by different water stresses faced by plants at different elevations.

Average inundation depth is an energy variable of flooding patterns. This value measures the relative energy magnitude of a flooding process assigned to different sites on the basis of elevation. For plants at lower surfaces, deep AID accompanied by high oxygen deficiency, high light deficiency, heavy mechanical damage, and other flooding stresses on physical parameters limits the production of vegetation. Thus, end-of-season biomass is negatively related to AID for sites with extremely deep inundation depth. In contrast, for plants in the higher surfaces, deep AID generally means good water availability and weakening drought stresses in their growth period. Thus, end-of-season biomass is positively related to AID for sites with extremely shallow inundation depth. Additionally, the differing hydrology to geomorphology processes in the lower and higher surfaces aggravates the response differences of end-of-season biomass to AID in different elevation zones. Specifically, erosion is the main hydro-geomorphology process in the lower surfaces (Lu et al., 2011; Wang et al., 2014). As a result, the soil at these elevations become more barren with a deeper AID, thus exacerbating the negative feedback of end-of-season biomass to AID in the lower surfaces. In contrast, deposition is the main hydro-geomorphology process in the higher surfaces. As a result, the deeper AID at these elevations indicates greater sedimentary nutrient accumulation, which exacerbates the positive feedback of end-of-season biomass to AID in the higher surfaces. Similar conclusions about the impact of flooding depth on community biomass have been confirmed by several studies (Seabloom et al., 1998; Wassen et al., 2002). For example, Lou et al. (2016) accessed the impact of flooding depth on wetland vegetation between different vegetation types and found that the biomass of *Carex lasiocarpa* residing at a higher elevation showed an increasing trend with increasing water-table depth, whereas *Carex angustifolia* residing at a lower elevation displayed a negative correlation between biomass and water-table depth.

The Gaussian form response of end-of-season biomass also applied to the flooding pattern variable IDU. IDU is a timing variable of flooding pattern, which is determined by both the start and end date of flooding events. Because both of the timing variables have different functions on plants at different elevations, the effect of IDU on end-of-season biomass also varies according to the distribution of plants. Specifically, for plants in the lower surfaces, the start time of inundation determines the end time of their first re-growth period before flooding. In contrast, for plants in the higher surfaces, the start time of inundation indicates the time at which they receive the first irrigation. The end time of flooding events determines the regeneration time of plants after flooding in the lower surfaces. Conversely, the end time of flooding events also determines when the plants begin to suffer from drought stresses for plants in the higher surfaces (Dai et al., 2019). Therefore, IDU determines the length of the growing period of plants in the lower surfaces, and the water availability of plants in the higher surfaces. Hence, end-of-season biomass is negatively correlated to IDU in the lower surfaces with long IDU and is positively correlated to IDU in higher surfaces with short IDU. Several results in previous studies have supported our conclusions. For example, Tan et al. (2015) revealed that *Carex* community residing at an elevation range of 13–15 m and a *Phragmites* community residing at an elevation range of 15–17 m had different optimum inundation durations, which were 159 days for the *Carex* community and 144 days for the *Phragmites* community.

Since the operation of the Three Gorges Dam, flooding of the Poyang Lake wetland has been significantly alleviated, along with the AID and IDU of the floodplain. According to a comparison of AID (IDU) between the two periods of 1980–2002 and 2003–2016, we estimated the variation of end-of-season biomass pre- and post-Three Gorges Dam through the obtained end-of-season biomass models established by AID (IDU). The results revealed an unbalanced variation of end-of-season biomass across the wetland. The end-of-season biomass decreased in higher surfaces because of the exacerbated drought stresses as the AID and IDU values decreased since 2003. The end-of-season biomass increased in lower surfaces because of the relief of flooding stresses as the AID and IDU values decreased. This conclusion is consistent with the results we obtained from the vegetation biomass study that examined land surface exposure time after flooding in the wetland (Dai, 2018). Importantly, different flooding variables, including AID and IDU, triggered different magnitudes of end-of-season biomass variation. The end-of-season biomass increase predicted by IDU was larger than that predicted by AID. We think this phenomenon can be attributed primarily to the higher adaptability of wetland vegetation to AID than its adaptability to IDU. For example, the study by Hu et al. (2018) in the Dongting Lake wetland revealed that the distribution elevations of wetland vegetation varied to some extent in different years, although their annual inundation duration was relatively constant. It also indicated that IDU may be a better indicator to predict changes in vegetation production in response to wetland flooding patterns.

In summary, this study explained the influence of flooding pattern variables on end-of-season biomass of the Poyang Lake wetland from the aspect of water stresses faced by plants residing at different elevations, thus providing insights into the effect of Three Gorges Dam on vegetation production of Poyang Lake wetland. Moreover, because a significant and continual alleviation of the flooding pattern after operation of the Three Gorges Dam is forecasted, a continual high level of vegetation production in the wetland can be expected. Hence, the additional impacts on wetland plants-dependent animals, including fish and migratory birds, should be further evaluated to maintain ecosystem stability and structural integrity of this important wetland, especially after the operation of the Three Gorges Dam.

## Data Availability Statement

All datasets generated for this study are included in the article/supplementary material.

## Author Contributions

XD, GY, and ZY conceived the study idea. XD and RW collected the data and conducted the relationship analysis between flooding patterns and end-of-season biomass. GY and ZY discussed and contributed to the final framework of this study. XD wrote the first draft of the manuscript with significant help from GY, ZY, and RW. All authors contributed to manuscript completion and revision.

## Conflict of Interest

The authors declare that the research was conducted in the absence of any commercial or financial relationships that could be construed as a potential conflict of interest.
